# Sambucus williamsii induced embryonic stem cells differentiated into neurons

**DOI:** 10.7603/s40681-015-0003-z

**Published:** 2015-02-02

**Authors:** Shih-Ping Liu, Chien-Yu Hsu, Ru-Huei Fu, Yu-Chuen Huang, Shih-Yin Chen, Shinn-Zong Lin, Woei-Cherng Shyu

**Affiliations:** 1 Center for Neuropsychiatry, China Medical University Hospital, 404 Taichung, Taiwan; 2 Graduate Institute of Basic Medical Science, China Medical University, 404 No. 91, Hsueh-Shih Road, Taichung, Taiwan; 3 Department of Social Work, Asia University, 413 Taichung, Taiwan; 4 Graduate Institute of Immunology, China Medical University, 404 Taichung, Taiwan; 5 Genetics Center, Department of Medical Research, China Medical University Hospital, 404 Taichung, Taiwan; 6 Graduate Institute of Chinese Medical Science, College of Chinese Medicine, China Medical University, 404 Taichung, Taiwan; 7 Department of Neurosurgery, China Medical University Beigan Hospital, 651 Yunlin, Taiwan; 8 Department of Neurosurgery, Tainan Municipal An-Nan Hospital-China Medical University, 709 Tainan, Taiwan

**Keywords:** Sambucus williamsii;, Differentiation;, neurons;, Embryonic stem

## Abstract

The pluripotent stem cells, including embryonic stem cells (ESCs), are capable of self-renewal and differentiation into any cell type, thus making them the focus of many clinical application studies. However, the efficiency of ESCs differentiated into neurons needs to improve. In this study, we tried to increase efficiently to a neural fate in the presence of various transitional Chinese medicines through a three-step differentiation strategy. From extracts of 10 transitional Chinese medicine candidates, we determined that Sambucus williamsii (SW) extract triggers the up-regulation of Nestin and Tuj1 (neuron cells markers) gene expression levels. After determining the different concentrations of SW extract, the number of neurons in the 200 μg/ml SW extract group was higher than the control, 50, 100, and 400 μg/ml SW extract groups. In addition, the number of neurons in the 200 μg/ml SW extract group was higher and higher after each time passage (three times). We also detected the Oct4, Sox2 (stem cells markers), Tuj1, and Nestin genes expression levels by RT-PCR. In the differentiated process, Oct4 and Sox2 genes decreased while the Tuj1 and Nestin genes expression levels increased. In summary, we demonstrated that SW could induce pluripotent stem cells differentiated into neurons. Thus, SW might become a powerful material for neurons–differentiating strategies.

## 1. Introduction

Embryonic stem cells (ESCs) have the ability to differentiate into ectodermal, endodermal, and mesodermal derivatives, giving them significant potential for clinical cell therapy applications. A lot of neurodegenerative diseases, including Parkinson’s disease and Alzheimer disease, do not have effective therapeutic methods. Cell therapy may become a powerful strategy to future clinical applications. However, neurons are hard to isolate and culture for clinical applications. For this reason, one of the sources to get neurons for future cell therapeutic applications for neurodegenerative diseases is ESC. Highly efficient approaches to differentiate ESCs into functional neurons are critical for modeling neurological disorders and testing potential therapies [1]. Previous studies have shown that a virus could be used to over-express neuralrelated genes to increase the differentiation efficiency [2, 3], but such a method is difficult to use in clinical applications. Protein transduction-based methods or small compounds can be efficient, safe alternatives to overcome this challenge [4]. In this study, we tried to discover if the extract of traditional Chinese medicine could increase the neurons-differentiation efficiency from ESCs. After we tested ten traditional Chinese medicines, we found that Sambucus williamsii had the potential to enhance neural-related genes (Nesin and TuJ1) expression levels. For this reason, we used Sambucus williamsii as our candidate extract to increase the differentiation efficiency of ESCs.

Sambucus williamsii is widely distributed in the North of China, Korea, and Japan [5]. In China, it has been used as a medicine for the treatment of bone and joint diseases for thousands of years [5]. Previous studies have shown that the compounds isolated from Sambucus williamsii can stimulate effects on osteoblastic UMR106 cell proliferation [6]. This research also showed that Sambucus williamsii could increase bone mass and bone strength in ovariectomized rats 7. However, the correlation between Sambucus williamsii and stem cells differentiation is unclear.

**Fig. 1:**
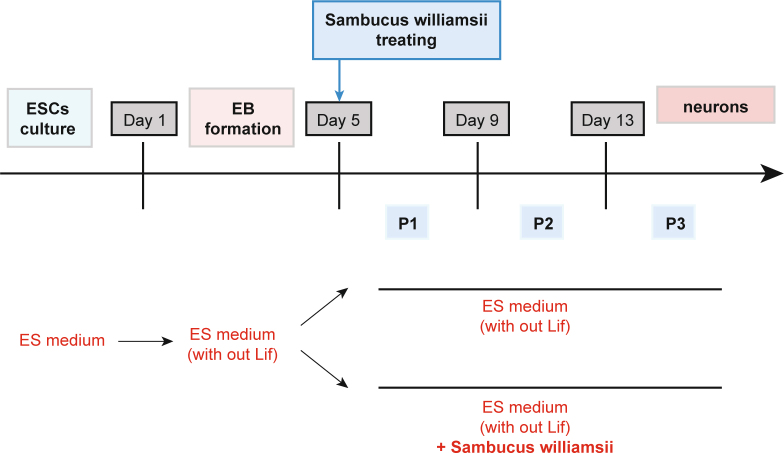
The three-step strategy to differentiate ESCs into neurons.

In this study, we used the ethanol extract of Sambucus williamsii to test the neurons differentiation ability of ESCs. We used a three-step differentiation strategy to differentiate ESCs into neurons (Figure 1). The genes expression levels of neural related genes were determined after Sambucus williamsii treatment by real-time PCR. After differentiating, immunefluoresce analysis and RT-PCR were used to test the optimal Sambucus williamsii concentrations for differentiating ESCs into neurons. In the end, we demonstrated that Sambucus williamsii could increase the neurons-differentiated efficiency of ESCs.

## 2. Materials and methods

### 2.1. Mouse embryonic fibroblast (MEF) cultures

Approval for all experimental protocols was applied for and received from the Institutional Animal Care and Use Committee of China Medical University. MEF isolation was performed as previously described [8]. Cells were collected from 13.5-dayold C57BL/6 mouse embryos retrieved by Cesarean section from mice purchased from the Taiwan National Laboratory Animal Center. Internal organs, legs, and heads were removed and remaining embryo parts digested with Trypsin. Cells were cultured in Dulbecco’s modified Eagle’s medium (DMEM) containing 10% heat-inactivated fetal bovine serum (FBS), penicillin (100 U/ml), streptomycin (100 μg/ml), non-essential amino acids (0.1 mM), and L-glutamine (2 mM) in a humidified incubator (37°C) with 5% CO_2_ (all chemicals and solutions from GIBCO BRL). MEF was treated with 10 μg/ml mitomycin C to become the feeder layers for the stem cell culture.

### 2.2. Mouse ESC cultures

Mouse ESCs (from the Taiwan Bioresource Collection and Research Center) were cultured in DMEM with 15% FBS (Hyclone, UT, USA), non-essential amino acids (0.1 mM), L-glutamine (2 mM), β-mercaptoethanol (0.2 mM) (GIBCO BRL), and LIF (4 ng/ml) (Millipore) in a humidified incubator at 37°C with 5% CO_2_.

### 2.3. The strategy of ESC differentiated into neurons

All three-step differentiation procedures were modified from standard protocols previously described [9, 10] and shown in Figure 1. Briefly, ESCs were transferred to Ultra-Low attached culture dishes in ES medium without LIF to generate embryoid bodies (EB). After 4 days, the embryoid bodies were transferred to normal dishes containing medium with or without the extract of Sambucus williamsii for neurons selection (P1). After 4 days, cells were subcultured to P2 generation. P3 generation also followed the same strategy. Immunefluoresces analysis was used for P1, P2, and P3 neurons.

### 2.4. Real-time PCR and reverse transcription PCR

Total RNA was extracted from ESCs, EB, P1, P2, and P3 neurons using TRIzol (Invitrogen) [11], with concentrations determined by spectrophotometry. Complementary DNA was produced from mRNA (5 mg) using a SuperScript III Reverse Transcriptase Kit (Invitrogen). Real-time PCR was used to determine Nestin and Tuj1 gene expression levels as previously described [11]. PCR conditions were predenatured at 94°C for 5 min followed by 28 cycles of amplification at 94°C for 30 s, 60°C for 30 s, and 72°C for 1 min, followed by a 10-min extension step at 72°C. PCR was performed with ExTaq (Takara) to detect the expression levels of the Oct4, Sox2, Tuj1, Nestin and β-actin genes.

### 2.5. Immunofluorescent (IF) antibody assays

IF antibody assays were performed as previously described [8]. Cultured cells were placed on slides, treated with fixing solution I (4% paraformaldehyde plus 400 mM sucrose in PBS) and held at 37°C for 30 min. Slides were then treated with fixing solution II (fixing solution I plus 0.5% Triton X-100) and held at room temperature for 15 min. After washing with PBS, slides were treated with a blocking buffer (0.5% BSA in PBS) at room temperature for 1 h and washed three additional times with PBS prior to reacting with various primary antibodies (anti-Nestin [abcam] or anti-Tuj1 [GeneTex]) at 1:100 dilutions, either overnight at 4°C or for 1 h at 37°C. Slides were washed five times with cold PBS before reacting with FITC-conjugated anti-mouse IgG or TRITCconjugated anti-rabbit IgG (Sigma-Aldrich). After four more washes with cold PBS, slides were mounted and observed using a fluorescence microscope. DNA was stained with DAPI (Sigma- Aldrich) to localize nuclei.

**Fig. 2:**
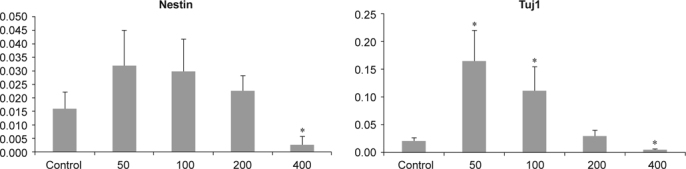
Nestin and Tuj1 gene expression levels in the P2 generation treated with various concentrations of Sambucus williamsii extract (real-time PCR).

**Fig. 3:**
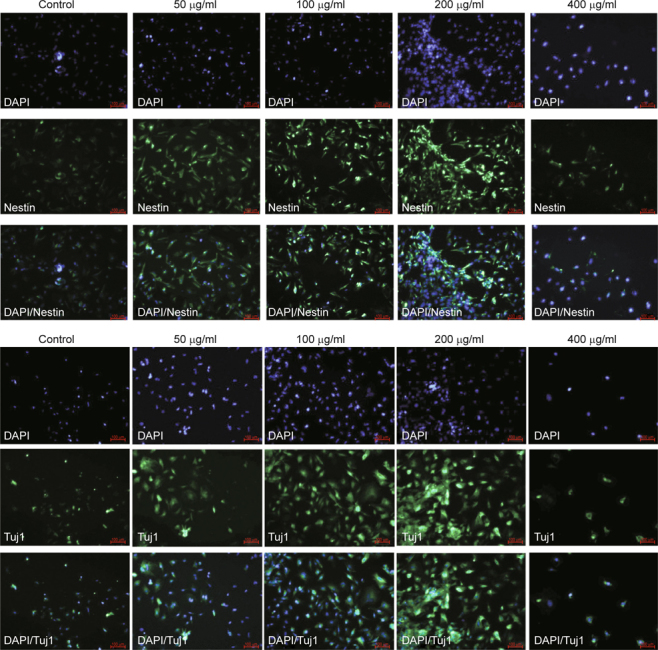
Nestin and Tuj1 expression levels after immunofluorescent staining in the P3 generation treated with various concentrations of Sambucus williamsii extract. Nuclei were stained with DAPI (blue).

**Fig. 4:**
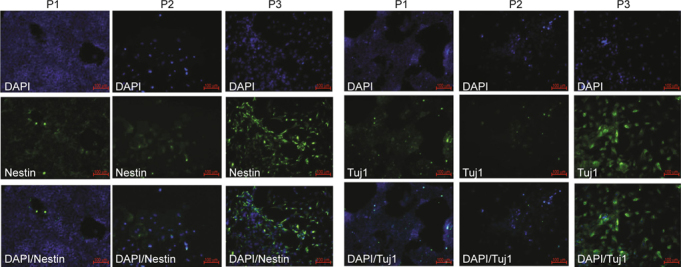
Comparison of Nestin and Tuj1 expression levels after immunofluorescent staining in the P1, P2 and P3 generations treated with various concentrations of Sambucus williamsii extract. Nuclei were stained with DAPI (blue).

## 3. Results

### 3.1. Neural related gene expression levels increased after Sambucus williamsii treatment

In order to determine that Sambucus williamsii could induce the neural related gene expression levels and increase the efficiency of ESCs differentiation into neurons, we measured Nestin and Tuj1 expression levels in the P3 generation after 50, 100, 200 and 400 μg/ml Sambucus williamsii treatment. As shown in Figure 2, Nestin expression levels increased after the 50, 100 and 200 μg/ml Sambucus williamsii treatment compared with the control group, especially in the 50 μg/ml. The Tuj1 expression levels also increased in the 50, 100 and 200 μg/ml Sambucus williamsii treatment group compared with the control group. This data indicates that Sambucus williamsii has the potential to induce the ESCs toward neuron differentiation.

### 3.2. Neural related protein expression levels increased after Sambucus williamsii treatment

After we determined the neural related genes expression levels increased after Sambucus williamsii treatment, we turned our attention to detecting the real differentiating ability of Sambucus williamsii. The differentiating strategy shown in Figure 1 and the P3 generation neurons was neural-related protein levels detected by IF analysis. In Figure 3, the results show that Nestin and Tuj1 protein expression levels in the 50, 100 and 200 μg/ml Sambucus williamsii treatment groups are higher than the control group and have the dose-response effect. Interestingly, Nestin and Tuj1 protein expression levels in the 400 μg/ml Sambucus williamsii treatment group were not higher than the control group. Nestin and Tuj1 expression mean the cells were neurons. Furthermore, this data shows the 200 μg/ml Sambucus williamsii treatment to have the optimal concentration to differentiate ESCs into neurons.

### 3.3. Neurons differentiation efficiency higher and higher though passage the cells

**Fig. 5:**
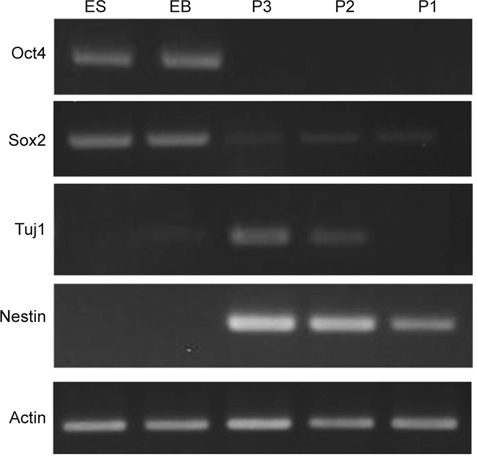
Gene expression levels of Oct4, Sox2, Tuj1, and Nestin in ESCs, EB, P1, P2 and P3 generations as measured by reverse transcription (RT)-PCR.

In our differentiating strategy, after passing through EB in normal dishes, cells cultured in a medium containing Sambucus williamsii passed to the P3 generation. If the Sambucus williamsii could induce ESCs to neurons, the percentage of neurons should increase during the passage times. In order to test this hypothesis, we used Nestin and Tuj1 as the neuron markers to detect the expression levels during the passage times. In Figure 4, Nestin and Tuj1 expression levels increased from the P1 to the P3 generation by IF assay. In addition, RT-PCR results also show that Nestin and Tuj1 gene expression levels increased from the P1 to the P3 generation. The whole stem cell markers’, Oct4 and Sox2, gene expression levels decreased during the P1 to the P3 generations (Figure 5). These data indicate that the medium containing Sambucus williamsii leads the ESCs toward neuron differentiation.

## 4. Discussion

Cell therapy will become a highly potential therapeutic method for many diseases that do not currently have efficient therapeutic methods, especially neurodegenerative diseases. However, neurons are hard to obtain and isolate. An efficient strategy to differentiate ESCs into neurons is very important in future cell therapy. Previous studies have shown that a virus could be used to over-express neural-related genes to increase the differentiation efficiency [2, 3] but such a method is hard to use for clinical applications. Protein transduction-based methods can be efficient, safe alternatives to overcome this challenge [4]. However, it is difficult for protein to cross cell membranes and so there is a need to use fusion proteins (Tat proteins [12-14], for example). Fusion proteins still have some problems that involve injuring cell membranes. For these reasons, small molecules may be a better candidate for neurons differentiation.

In this study, we used a three-step differentiation strategy to determine that Sambucus williamsii could induce ESCs to differentiate into neurons. Most of studies have shown the application of Sambucus williamsii in bone and osteoblastic research [6, 7]. This is the first study to investigate the correlation between Sambucus williamsii and stem cells differentiation. In the end, we are confident we found a new applicationfor Sambucus williamsii.

In conclusion, this study discovered a traditional Chinese medicine that could potentially be used to increase the differentiation efficiency of ESCs into neurons. In addition, a simple method to differentiate ESCs into neurons was also developed. It should prove helpful in future neurons therapy from the pluripotent stem cells, including induced pluripotent stem (iPS) cells. We hope it will also be helpful in the future clinical application of cell therapy.

### Acknowledgements

This study is supported in part by the Taiwan Ministry of Health and Welfare Clinical Trial and Research Center of Excellence (MOHW104-TDU-B-212-113002), and China Medical University (CMU101-NSC-06).
